# Heterogeneity induces rhythms of weakly coupled circadian neurons

**DOI:** 10.1038/srep21412

**Published:** 2016-02-22

**Authors:** Changgui Gu, Xiaoming Liang, Huijie Yang, Jos H. T. Rohling

**Affiliations:** 1 Business School, University of Shanghai for Science and Technology, Shanghai 200093, China; 2 School of Physics and Electronic Engineering, Jiangsu Normal University, Xuzhou 221116, China; 3 Department of Molecular Cell Biology, Laboratory for Neurophysiology, Leiden University Medical Center, Leiden 2300RC, The Netherlands

## Abstract

The main clock located in the suprachiasmatic nucleus (SCN) regulates circadian rhythms in mammals. The SCN is composed of approximately twenty thousand heterogeneous self-oscillating neurons, that have intrinsic periods varying from 22 h to 28 h. They are coupled through neurotransmitters and neuropeptides to form a network and output a uniform periodic rhythm. Previous studies found that the heterogeneity of the neurons leads to attenuation of the circadian rhythm with strong cellular coupling. In the present study, we investigate the heterogeneity of the neurons and of the network in the condition of constant darkness. Interestingly, we found that the heterogeneity of weakly coupled neurons enables them to oscillate and strengthen the circadian rhythm. In addition, we found that the period of the SCN network increases with the increase of the degree of heterogeneity. As the network heterogeneity does not change the dynamics of the rhythm, our study shows that the heterogeneity of the neurons is vitally important for rhythm generation in weakly coupled systems, such as the SCN, and it provides a new method to strengthen the circadian rhythm, as well as an alternative explanation for differences in free running periods between species in the absence of the daily cycle.

Circadian rhythms in behavior and physiology are regulated by the main clock which is located in the suprachiasmatic nucleus (SCN) in mammals^1^,^2^,^3^,^4^. The SCN has two main functions. Firstly, the SCN functions as a rhythm generator with a period close to but not exactly 24 h^5^,^6^. In the absence of a light-dark cycle, our body is entrained to this so-called ‘free-running’ rhythm in behavior. The periods of the free running rhythm vary between species, for example 24.2 h for a human being, 22.9 h for a deer mouse, and 23.8 h for a southern flying squirrel^3^. In addition to this endogenous rhythm, the SCN receives periodic light information of the external light-dark cycle and synchronizes the bodily rhythms to this external daily cycle, creating a period of exactly 24 h^7^,^8^.

The SCN is a heterogeneous structure, consisting of two nuclei located just above the optic chiasm in the hypothalamus, on opposite sides of the third ventricle. Each nucleus contains approximately 10,000 self-oscillating neurons with heterogeneous intrinsic periods ranging from 22 h to 28 h^9^,^10^,^11^. Furthermore, each nucleus is divided roughly into two distinct functional subgroups, i.e. a light-sensitive subgroup, which receives direct photic input from the retina, is located in the ventrolateral SCN (VL), and consists of approximately 25% of the SCN neurons in rats^12^,^13^. The second group is not directly light-sensitive and we will call these the dorsomedial subgroup (DM)^13^,^14^,^15^,^16^.

The heterogeneous neurons are coupled through different neurotransmitters and neuropeptides. The VL and DM subgroups are coupled through a neurotransmitter called γ-aminobutyric acid (GABA) which is abundantly present throughout the SCN. The coupling is asymmetrical (heterogeneous) between the VL and DM, because the VL dominates the DM, but the DM also feeds back to the VL^15^,^16^,^17^. Within these regions different neurotransmitters and neuropeptides are used for communication between the cells, for example the VL neurons express vasoactive intestinal polypeptide (VIP) and the DM neurons produce arginine vasopressin (AVP). Other coupling mechanisms, such as gap-junctions, make this a heterogeneously coupled network. Nevertheless, this whole network of heterogenous neurons which are heterogeneously coupled achieves a uniform network period^18^.

The coupling within the SCN is not considered to be very strong. Strong coupling implies complete synchrony between the cells, but in the SCN the cells are only partially synchronized as the periods of the cells deviate even in the intact SCN^19^. The heterogeneity and the weak coupling were thought to weaken the circadian rhythms of the SCN^8^,^20^,^21^,^22^,^23^,^24^. Previous studies found that the heterogeneity of the neurons and of the network reduces both the amplitude of the circadian rhythms of the single cells and the synchronization degree between the neuronal oscillators, and it also decreases the period of the SCN network^20^,^21^,^22^. In addition, weak coupling is shown to result in the attenuation of the circadian rhythms experimentally in aged mice^24^ or based on theoretical models^8^,^23^.

However, the effect of heterogeneity on the collective behavior of SCN neurons is still not well understood for weak coupling conditions. In the present study, we examined the effect of neuronal heterogeneity on the collective behavior of the SCN neurons based on the Goodwin model using mean field coupling^20^. In this model the SCN neurons are heterogeneous in their endogenous periods which are normally distributed around a mean. Furthermore, we added heterogeneity to the network be introducing heterogeneity in the coupling between the neuronal subpopulations in the VL and DM SCN. We found that for weak coupling the neuronal heterogeneity induces oscillations and strengthens the collective circadian rhythm, which is in contrast to strong coupling. Homogeneous neurons do not show this result when they are weakly coupled. An increase in network heterogeneity results in decreased amplitude of the SCN rhythm, but does not affect the dynamical behavior experienced for neuronal heterogeneity. In the following sections we will introduce the Goodwin model in the Methods section, the effect of heterogeneity and the theoretical analysis in the Results section, and finally we discuss our findings.

## Methods

The Goodwin oscillator is widely used to model SCN neurons^20^,^22^,^23^,^25^,^26^,^27^,^28^. One single neuronal oscillator is represented by a negative feedback loop which is formed by the mRNA of a gene (*X*), its protein product (*Y*) and an inhibitor of that gene (*Z*). The oscillators are coupled through a mean field of a certain neurotransmitter, in this case GABA. The Goodwin model composed of *N* coupled neurons is described as:


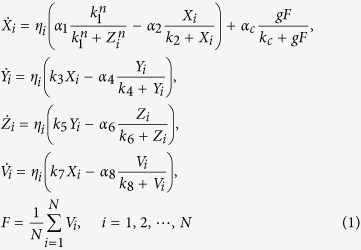


where there are four variables in each neuron *i*, i.e. the gene mRNA *X*_*i*_, the protein *Y*_*i*_, the inhibitor *Z*_*i*_, and the neurotransmitter release *V*_*i*_. The amount of neurotransmitter *V*_*i*_ that is produced depends upon the gene mRNA *X*_*i*_. The mean value for the released neurotransmitter *V* for all neurons is represented by the mean field *F*. The sensitivity of the oscillator to this mean field is represented by coupling strength *g*. In the present study, *V*_*i*_ and *F* represent the oscillatory output of one individual neuronal oscillator and of the SCN network respectively.

The heterogeneity in this model is imposed both on the neuronal level as well as on the network level. To model the heterogeneity of the network, asymmetrical coupling strengths for the VL and DM group were examined. This will be explained later in this Methods section. On the neuronal level, heterogeneous intrinsic periods of neurons are established by introducing the factor *η*_*i*_^20^, which affects all variables, as can be seen in Eq. (1), where it is included in all equations for *X*, *Y*, *Z*, and *V*. The factors *η*_*i*_ satisfy a normal distribution with a unit mean and a standard deviation *δ*, where *δ* is the degree of heterogeneity.

In the present study, we only investigated the endogenous network rhythmicity without external input. For this, we assessed the free running behavior of the SCN in the absence of an entraining light-dark schedule. This means that there is no light input term to both the VL and the DM in Eq. (1). When *N* = 1, the transmitter *V*_*i*_ is produced and absorbed by neuron *i* itself, thus the neuron oscillates with its own intrinsic period. The values of other parameters are set as in ref. 23: *α*_1_ = 6.8355 nM/h, *k* = 2.7266 nM, *n* = 5.6645, *α* = 8.4297 nM/h, *k*_2_ = 0.2910 nM, *k*_3_ = 0.1177/h, *α*_4_ = 1.0841 nM/h, *k*_4_ = 8.1343 nM, *k*_5_ = 0.3352/h, *α*_*6*_ = 4.6645 nM/h, *k*_*6*_ = 9.9849/h, *k*_*7*_ = 0.2282/h, *α*_*8*_ = 3.5216 nM/h, *k*_*8*_ = 7.4519 nM, *α*_*c*_ = 6.7924 nM/h, *k*_*c*_ = 4.8283 nM.

Three characteristics of the collective behavior of the SCN neurons have been studied, i.e. the amplitude and the period of mean field *F*, and the synchronization between the neuronal oscillators. The network amplitude *ρ* is defined as:


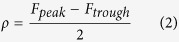


The synchronization degree *R* between the neuronal oscillators is measured over time as in refs 20,23:


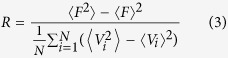


where 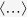 represents average over time. *R* is 0 for fully desynchronized oscillators and 1 for perfect synchronization. As reported in refs 22,23, the synchronization degree *R*, the period *T* and the amplitude *ρ* of the network are governed by the coupling strength *g*. In the supplementary Fig. S1, the relationship between the network amplitude *ρ* and the coupling strength *g* is examined. A Hopf bifurcation can be seen when *g* = 0.8. When *g* > 0.8, the SCN network oscillates with high amplitude. When *g* ≤ 0.8, the SCN network loses its rhythm as the amplitude reduces to zero (Fig. S1). We define *g* = 0.8 as a critical transition point between weak and strong coupling, where we call *g* > 0.8 strong coupling and *g* ≤ 0.8 weak coupling. In this study we investigated the effects of the heterogeneous degree *δ* on the network amplitude *ρ*, synchronization degree *R* and network period *T* for strong coupling with *g* = 1.0, 0.95, 0.9, 0.85, and 0.81 and weak coupling with *g* = 0.80, 0.79, 0.78, 0.77, and 0.76 using Eq. (1).

In addition to neuronal heterogeneity, we introduce heterogeneity in the network by adding asymmetrical coupling between the group of VL neurons and the group of DM neurons^15^,^16^,^17^. We introduced an additional coupling parameter 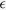
_*i*_ for each neuron, which differs between VL and DM neurons. We ensure that the coupling from the VL group to the DM group becomes larger than from DM to VL. Eq. (1) is modified as follows:


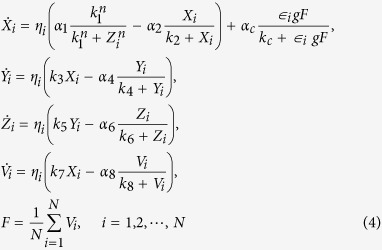


where the coupling strength is determined by *ε*_*i*_*g*, satisfying 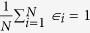. Let 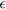
_*i*_* g* = *G*_*a*_ if oscillator *i* is in the VL group (1 ≤ *i* ≤ *N*_*a*_), and 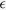
_*i*_* g* = G_*b*_ when oscillator *i* is in the DM group (*N*_*a*_ < *i* ≤ *N*). In this study, *N*_*a*_ is set as 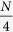, since the number of neurons in the light-sensitive VL group is approximately 25% of the number of SCN neurons^12^,^13^. As VL has a larger effect on DM than vice versa, the mean field affects the DM group more than the VL group, thus *G*_*a*_ ≤ *G*_*b*_, in our model. *κ* is introduced as a factor for the relative difference between *G*_*a*_ and *G*_*b*_, where 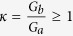. If *κ* = 1, we have the uniform network, as was described by Eq. (1). If *κ* *>* *1*, *G*_*b*_ > *G*_*a*_, so the neurotransmitter *F* has a larger influence on DM neurons than on VL neurons.

We test the influence of *κ* on the rhythmic network output. It is apparent that the coupling of DM and VL changes if *κ* *>* *1*. In supplemental Fig. S2 we show for weak coupling (*g* = 0.79) that if *κ* > 1.05 the coupling strengths *G*_*a*_ and *G*_*b*_ run outside the boundaries of the weak coupling region 0.76 < *g* ≤ 0.80 (see Analytical part). The DM oscillators become strongly coupled, while the VL oscillators become weakly coupled. Thus, to ensure that weak coupling is present everywhere in the SCN, we restrict ourselves to values of 1 ≤ *κ* < 1.05. For these values we examined the effect of the heterogeneity parameter *κ* for the network on the amplitude *ρ* of the network, on the synchronization degree *R* between the neuronal oscillators and on the period *T* of the network, for strong and weak coupling respectively.

The fourth-order Runge-Kutta method was used for numerical simulations with time increments of 0.01 h. The initial 5,000,000 time steps (50,000 h) were neglected in order to avoid the influence of transients. The number of neurons *N* was set to 500 for the numerical simulations. The initial conditions for each variable were selected randomly from a uniform distribution in the range [0–1] for *X*, *Y*, *Z*, and *V*. We also chose *N* = 100 and *N* = 1000, and found that the results were in accordance with the results of *N* = 500.

## Results

### The effect of the heterogeneity of neurons

**Figure 1:**
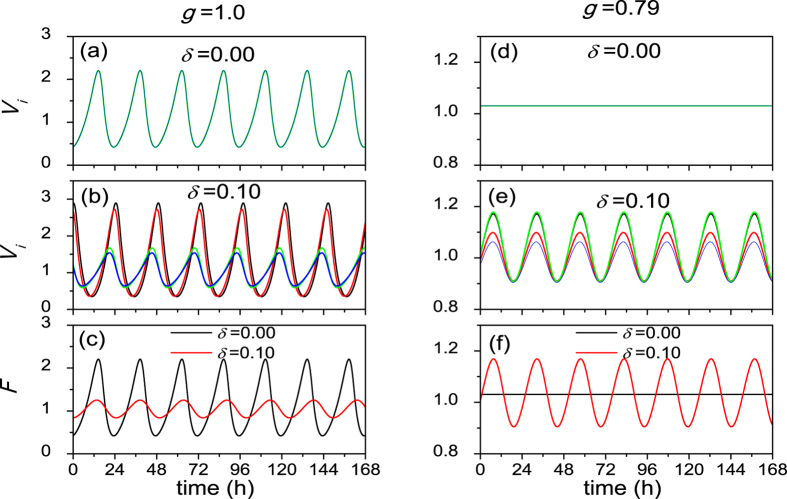
The evolutions of neuronal oscillators when they are homogeneous or heterogeneous, in the case of strong coupling with *g = *1.0 (**a**–**c**) and in the case of weak coupling with *g* = 0.79 (**d**–**f**). Evolutions of two randomly chosen oscillators in the VL (described by the black and red lines) and the DM (described by the blue and green lines) with the heterogeneity degree *δ* = 0.0 (**a**–**d**), and with the heterogeneity degree *δ* = 0.1 (**b**–**e**). (**c**–**f**) The comparison of the evolutions of the SCN network (mean field *F*) for homogeneous oscillators (*δ* = 0) and heterogeneous oscillators (*δ* = 0.1) for strong (**c**) and weak (**f**) coupling.

Typical traces for individual cells and the SCN network are presented in Fig. 1, for strong coupling (*g* = 1.0) and weak coupling (*g* = 0.79). As reported in refs. 23,22, in the case of strong coupling, the neurons oscillate with remarkable amplitude and maintain full synchrony when the neurons are homogeneous i.e. *δ* = 0.0 (a). With the increase of the neuronal heterogeneous degree *δ*, the amplitude of VL oscillators increases and of the DM oscillators decreases. Furthermore, the phase of the DM oscillators is advanced (b). In (c) a decrease of the amplitude of the SCN network is observed for increased neuronal heterogeneity. Moreover, the peak is delayed under *δ* = 0.1 because the period of the network is longer under *δ* = 0.1 than that under *δ* = 0.0. In the case of weak coupling, when the neurons are homogeneous (*δ* = 0.0), the amplitude of the neuronal oscillators is 0 (d). Interestingly, in the presence of the heterogeneity with *δ* = 0.1, individual neurons show an oscillation with remarkable amplitude and both the VL and DM oscillators remain highly synchronized (e). We could not observe a rhythm for the SCN (meaning that the SCN amplitude is zero) when *δ* = 0.0 due to the zero amplitude of the individual neurons, but we observed a robust oscillation of the network with *δ* = 0.1 in (f).

**Figure 2:**
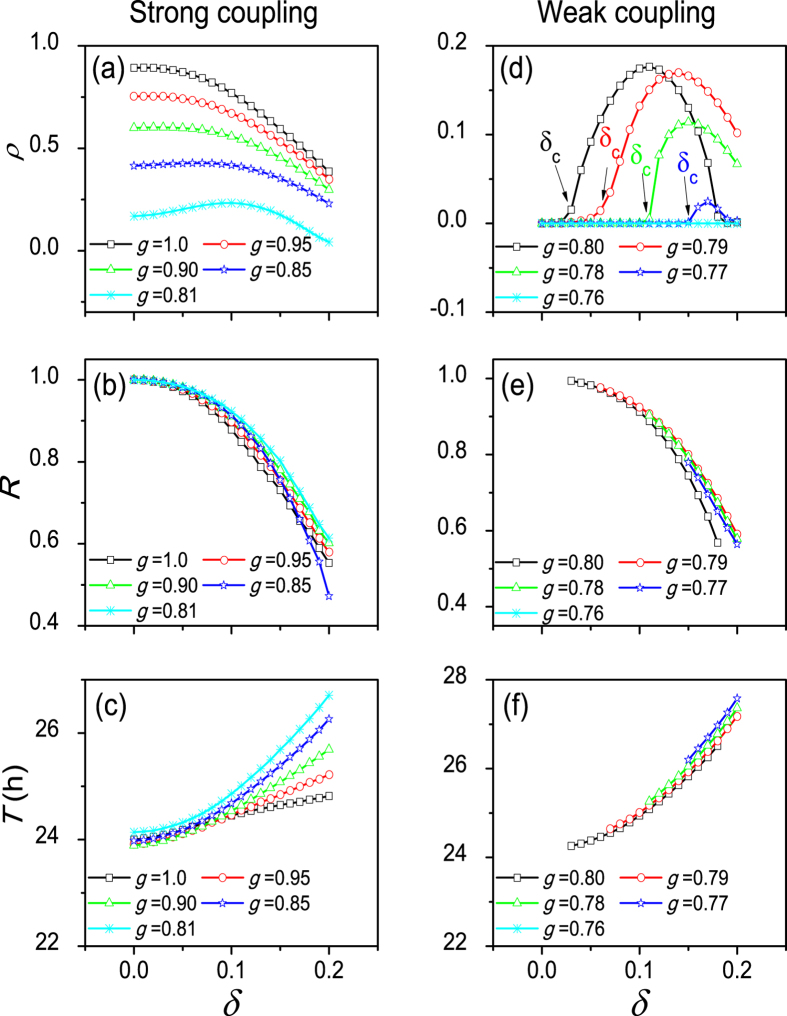
The dependence of the collective behavior parameters on the heterogeneous degree *δ*, in the cases of strong coupling with *g* = 1.0,095, 0.90, 0.85 and 0.81 (**a**–**c**), and in the cases of weak coupling with *g* = 0.80,0.79, 0.78, 0.77 and 0.76 (**d**–**f**). Panels (**a**,**d**) show the relationship between the network amplitude *ρ* and the neuronal heterogeneity degree *δ*, panels (**b**,**e**) show the relationship between the synchronization degree *R* between the neuronal oscillators and *δ* and panels (**c**,**f**) show the relation between period *T* and *δ*.

The dependence of the parameters of the collective behavior on the heterogeneous degree *δ* is shown in Fig. 2 for strong coupling with *g* = 1.0, 0.95, 0.9, 0.85, and 0.81 and weak coupling with *g* = 0.80, 0.79, 0.78, 0.77, and 0.76. For strong coupling, an increase of *δ* decreases the amplitude *ρ* of the SCN network (a), as well as the synchronization degree *R* between neuronal oscillators (b). The period *T* of the SCN network increases with the increase of *δ* (c). For weak coupling, small values of *δ* do not show a rhythm amplitude (*ρ* = 0) (d). When *δ* is larger than a critical value *δ*_*c*_, an oscillation appears with an amplitude *ρ* > 0, where *δ*_*c*_ is 0.03, 0.08, 0.12 and 0.15 for *g* = 0.8, 0.79, 0.78, and 0.77 respectively. With coupling *g* = 0.76, the absence of *δ*_*c*_ and the zero amplitude of *ρ* are independent of *δ*, which is also true for all values of *g* < 0.76. Consistent with the results for strong coupling, an increase of *δ* decreases the synchronization degree *R* (e) and increases the network period *T* (f). Note that, in the case of *g* ≤ 0.76, and for other cases of weak coupling when *δ* < *δ*_*c*_, the network amplitude *ρ* and the amplitude of individual neurons are 0, thus the calculation of *R* and *T* is not possible. This means that the heterogeneity of neurons can induce an oscillation in single neuronal oscillators and strengthen the circadian rhythm (increase the amplitude) of the SCN network when *δ* ≥ *δ*_*c*_ in the cases of *g* = 0.80, 0.79, 0.78, and 0.77.

### The effect of the heterogeneity of the network

Heterogeneity of the network may be a result from the topology or structure of the network, but it may also result from heterogeneous coupling between (groups of) nodes in the network. Because the network organization is directly related to coupling between the neurons, and the coupling between the VL and DM SCN is not symmetrical^15^,^16^,^17^, we investigate in this study network heterogeneity resulting from the difference of coupling between the VL and DM SCN.

**Figure 3:**
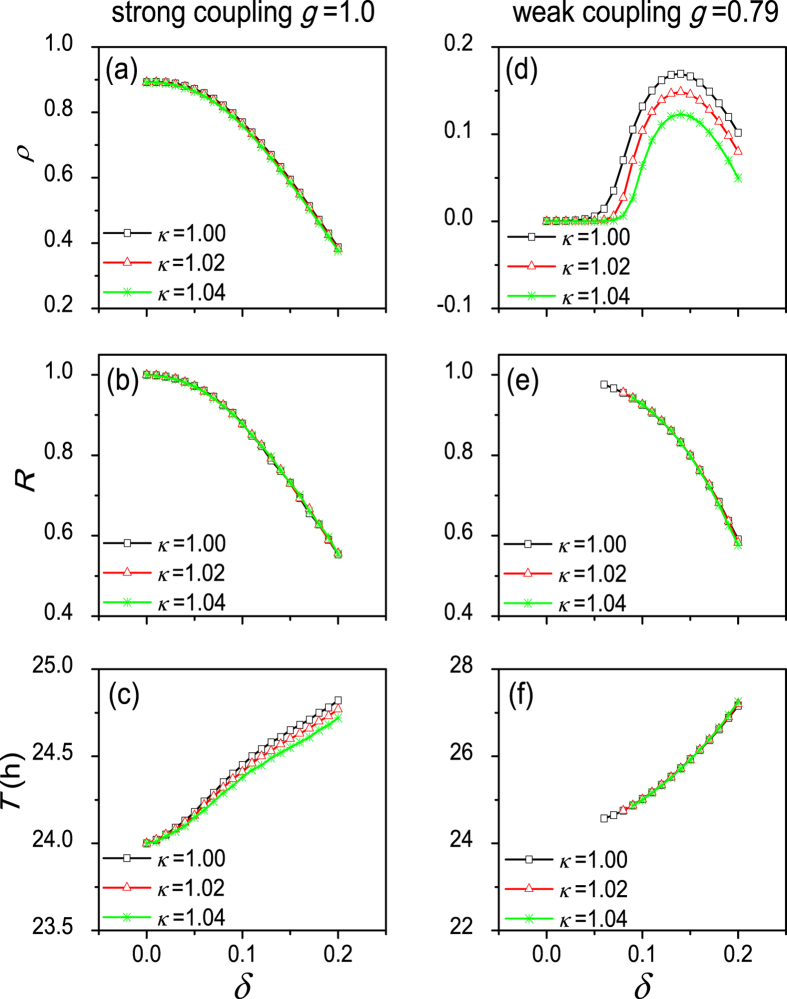
The dependence of the collective behavior parameters on the heterogeneous degree *δ*, depending on network heterogeneity parameter *κ*. The parameter *κ* represents the ratio of coupling strength of the DM to coupling strength of the VL. Similar to Fig. 2 we show the relationship between the amplitude *ρ* of the SCN network and *δ* (**a**–**d**), the relationship between the synchronization degree *R* and *δ* (**b**–**e**) and the relationship between the period *T* of the SCN network and *δ* (**c**–**f**).

Adding this network heterogeneity to the model does not change the characteristics of the network seen for neuronal heterogeneity. However, an increased heterogeneity in *κ* negatively affects the maximum value of network amplitude *ρ*. No noticeable difference in synchronization degree *R* and in network period *T* are found when the network heterogeneity increases (Fig. 3).

### Analytical results

To examine the transition point that we see with weak coupling, we analytically investigate how the oscillation, that is induced by the heterogeneity of the neuronal oscillators, emerges. With the increase of the degree *δ*, the disappearance of zero amplitude and the emergence of a limit cycle suggest that there is a Hopf bifurcation (Figs 2 and S2). In order to investigate the existence of the Hopf bifurcation, we must perform three steps, i.e. find the Jacobian matrix of Eq. (1), search the equilibrium points of Eq. (1) against the parameter *δ* and examine the stability of the equilibrium points against the parameter *δ*^29^,^30^. For analytical simplicity, let the number of neurons be *N* = 2, and let ‘*a*’ and ‘*b*’ represent one neuron in the VL and the other one in the DM respectively. The factors *η*_*i*_ are set as *η*_*a*_ = 1 − *δ* and *η*_*b*_ = 1 + *δ*. The Jacobian matrix of Eq. (1) with two coupled oscillators is shown in Eq. (5):


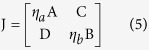


where


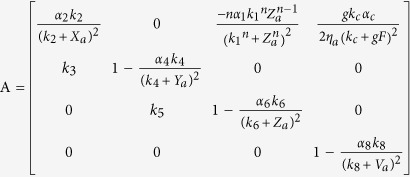



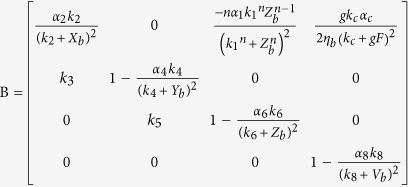



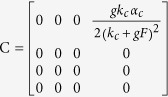



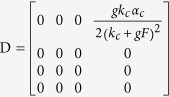


Next, we look for the equilibrium points against the heterogenous degree *δ*. Letting 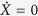, 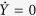, 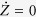, 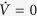, Eq. (1) with *N* = 2 can be written as:


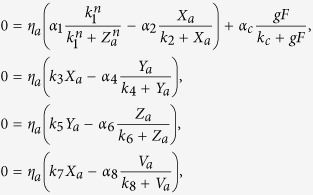



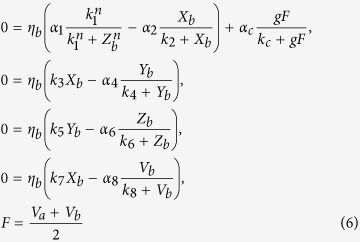


**Figure 4:**
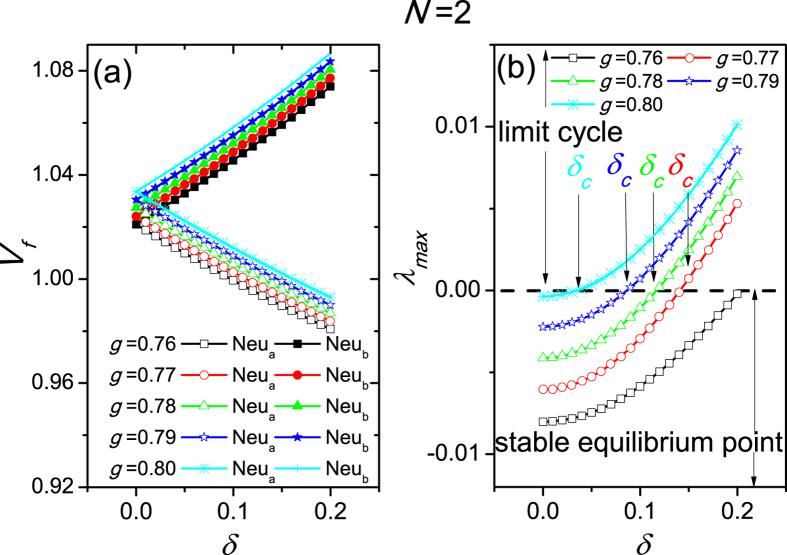
The stability analysis of the equilibrium points in the cases of weak coupling with *g* = 0.76,0.77,0.78,0.79 and 0.80. (**a**) The dependence of the equilibrium points on the heterogeneous degree *δ*. (**b**) The (un)stability of the equilibrium points. The dashed line *λ*_*max*_ = 0 divides the plane into two regions, in one of which the equilibrium points are stable due to *λ*_*max*_ < 0, and in the other of which the equilibrium points are unstable due to *λ*_*max*_ ≥ 0 and a limit cycle oscillation emerges. *λ*_*max*_ is equal to 0, when *δ* = *δ*_*c*_. *g* represents coupling strength, and Neu *a*(*b*) stands for Neuron *a*(*b*).

We use numerical simulation to find the equilibrium points of Eq. (6) against the heterogenous degree *δ*. The dependence of the equilibrium points on *δ* with weak coupling is shown in Fig. 4(a). After the equilibrium points have been found numerically, we examine the stability of these equilibrium points. If the equilibrium point is stable, the amplitude of the oscillations becomes 0; if the equilibrium point is unstable, a limit cycle (oscillation) appears. The Hopf bifurcation appears at the transition from the stable equilibrium point to the unstable equilibrium point. The (un)stability is determined by the maximal value *λ*_*max*_ of the eigenvalues’ real parts of the Jacobian matrix of Eq. (1). If *λ*_*max*_ < 0, the equilibrium point is stable, which corresponds to a situation where there is no rhythm in the neurons (amplitude is 0) and thus to a loss of rhythm in the SCN; if *λ*_*max*_ ≥ 0, the equilibrium point is unstable which corresponds to rhythmic ‘limit cycle’ behavior. The dependence of *λ*_*max*_ on *δ* is shown in Fig. 4(b). The dashed line *λ*_*max*_ = 0 divides the investigated plane into two regions, i.e. the region of ‘limit cycle’ oscillatory behavior and the region of ‘stable equilibrium point’, where oscillations are absent. When the coupling is *g* = 0.80, 0.79, 0.78, and 0.77, a Hopf bifurcation marks the point of the critical transition (*δ*_*c*_) between oscillatory and non-oscillatory behavior. In these cases, we have *λ*_*max*_ < 0 when *δ* *<* *δ*_*c*_, and *λ*_*max*_ ≥ 0 when *δ* ≥ *δ*_*c*_. The values of *δ*_*c*_ are 0.04, 0.09, 0.12 and 0.15 for *g* = 0.80, 0.79, 0.78, and 0.77 respectively, which are very close to the values of *δ*_*c*_ in Fig. 2(d). On the other hand, with coupling *g* = 0.76, the *δ*_*c*_ and the Hopf bifurcation are absent, as the line is always below *λ*_*max*_. For strong coupling, with *g* > 0.80, the line is always above *λ*_*max*_ indicating that the oscillatory condition is always present. Therefore, we have proven that for weak coupling with *g* in the range of [0.77, 0.80], heterogeneity of neurons induces the neuronal oscillation when *δ* > *δ*_*c*_.

## Discussion

In this study, we investigated the role of heterogeneity of the neurons and of the network in the collective behavior of the SCN neurons. We found that there are distinct roles for neuronal heterogeneity in case of strong coupling and weak coupling. In case of strong coupling, the neuronal heterogeneity reduces the amplitude of the network. In contrast, in the of weak coupling, the neuronal heterogeneity induces the oscillation and strengthens the circadian rhythm. In both cases, the synchronization between the neurons is disturbed and the period of the network is increased.

The heterogeneity in the coupling of the network through asymmetrical coupling between VL and DM parts of the SCN does not affect the network oscillatory behavior, but it does affect the maximum amplitude of the network for weak coupling. A homogeneous network, where the coupling between the VL and DM parts of the SCN are the same leads to a higher maximum amplitude of the network than a heterogeneously coupled network. However, the dynamics in the network is not affected by the heterogeneity in the coupling of the network, but seems only determined by the heterogeneity of the neurons.

The network heterogeneity that we investigated stems from neurotransmitter coupling through GABA. We investigated the GABA-ergic coupling because the majority of fast connections between SCN neurons is mediated through the GABA_A_ receptor^31^. GABA can be excitatory or inhibitory depending on the chloride concentration in the cell^32^. Excitatory and inhibitory action of GABA can both increase or decrease synchrony among cells^31^,^33^,^34^,^35^,^36^,^37^. Furthermore, other neurotransmitters and neuropeptides also promote synchronization, such as VIP in the ventral SCN^36^. The interplay between excitatory and inhibitory effects and their influences on either synchronization and desynchronization as active processes^31^ is very complex and at least partly reported in ref. 38. Here, we focused on the synchronizing effect of GABA, but we believe that this complex interplay of synchronizing and desynchronizing effects is an important direction for future research.

In addition to heterogeneity in coupling within the network, the network topology also plays an important role in the network behavior of the SCN. The topology we considered here was an all-to-all coupling between the neurons, which is not a very realistic topology for the SCN network. The SCN network is assumed to be scale free^39^,^40^ and to possess small world properties^41^,^42^. For example, a small world network has been shown to provide more precise circadian rhythms, a larger amplitude, higher synchrony and shifts more rapidly after introduction of a new light-dark regime compared to a random or local connection network^40^,^42^,^43^,^44^,^45^. However, these studies only considered the case of strong coupling. Compared to strong coupling, we recommend further research investigating the influence of neuronal network topologies on the rhythmic properties of the SCN in the case of weak coupling.

The present study shows that for weak coupling, the heterogeneity of the neurons is of major importance for the coordinated output of the neurons. A weakly coupled system, such as the SCN, should have neuronal heterogeneity to achieve a rhythmic output altogether. The finding that the SCN period is positively related to the heterogeneous degree of the neurons may provide an insight to the variance of the free running periods between species^3^. Also, as the range for weak coupling is very narrow, a small decrease in coupling strength disrupts the circadian rhythms in mice, as can be seen experimentally in aging^24^. And finally, this study shows that the heterogeneity of the neurons induces oscillations and increases the SCN amplitude, which may provide a novel method for strengthening the circadian rhythms in aging. We hope our study also helps understanding the synchronization phenomenon in other neuronal systems^46^,^47^,^48^.

## Additional Information

**How to cite this article**: Gu, C. *et al.* Heterogeneity induces rhythms of the weakly coupled circadian neurons. *Sci. Rep.* **6**, 21412; doi: 10.1038/srep21412 (2016).

## Supplementary Material

Supplementary Information
