# Supplementary material for: Heterogeneity induces rhythms of weakly coupled circadian neurons
Source: Sci Rep. 2016 Feb 22;6:21412. doi: 10.1038/srep21412 (PMC4761972; doi:10.1038/srep21412)
Supplement: Supplementary Information [file srep21412-s1.pdf]

Heterogeneity induces rhythms of the weakly coupled circadian neurons

Changgui Gu[1*], Xiaoming Liang[2], Huijie Yang[1], and Jos Rohling[3*]

[1]Business School, University of Shanghai for Science and Technology, Shanghai 200093, China

[2]School of Physics and Electronic Engineering, Jiangsu Normal University, Xuzhou 221116, China

[3]Department of Molecular Cell Biology, Laboratory forNeurophysiology, Leiden University Medical Center, Leiden, The Netherlands

*Corresponding author: gu_changgui@163.com; j.h.t.rohling@lumc.nl

Supplementary Information

The relationship between the network amplitude and the coupling strength

The relationship between the network amplitude ρ and the coupling strength g is shown in Fig S1, when the oscillators are homogeneous with $\delta = 0.0$. There is a critical point $g = g_c = 0.8$ which separates the investigated region into two parts, i.e. when $g > g_c$ the amplitude $\rho > 0$ and when $g \le g_c$ the amplitude $\rho = 0$. Thus, we define strong coupling as $g > g_c$ and weak coupling as $g \le g_c$.

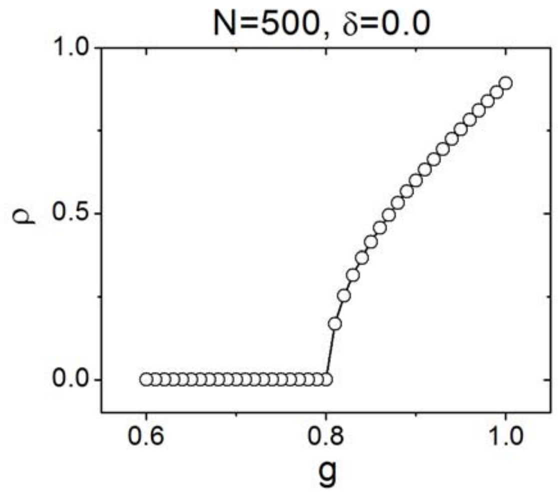

Figure S1 | The relationship between the network amplitude ρ and the coupling strength g when the oscillators are homogeneous with $\delta = 0.0$. The number of oscillators is represented by N.

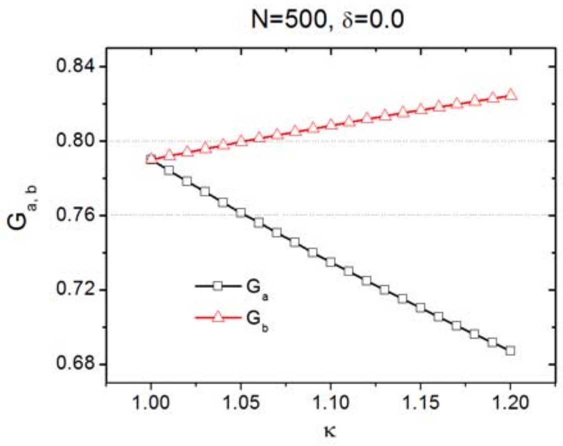

Figure S2 | The relationship of the coupling strength of VL neurons G_a and the coupling strength of DM neurons G_b to the ratio κ when the oscillators are homogeneous with $\delta = 0.0$. The number of oscillators is represented by N. The region between the two dotted lines is the region of weak coupling.
